# The evolving role of the renin–angiotensin system in ARDS

**DOI:** 10.1186/s13054-017-1917-5

**Published:** 2017-12-28

**Authors:** Eleni Vrigkou, Iraklis Tsangaris, Stefanos Bonovas, Argyrios Tsantes, Petros Kopterides

**Affiliations:** 1 2nd Department of Critical Care Medicine, Attikon University Hospital, National and Kapodistrian University of Athens, Rimini Str. 1, Athens, 12462 Greece; 2 Department of Biomedical Sciences, Humanitas University, Milan, Italy; 3 Humanitas Clinical and Research Center, Milan, Italy; 4 Laboratory of Hematology and Blood Bank Unit, Attikon University Hospital, Athens, Greece

We read with great interest the article by Khan et al. [1] reporting the results of a pilot clinical trial of administrating recombinant angiotensin-converting enzyme 2 (ACE2) in patients with acute respiratory distress syndrome (ARDS). Current research has been focused on the involvement of the renin–angiotensin system (RAS) in the pathogenesis and clinical outcomes of ARDS and, in particular, in the evaluation of the angiotensin-converting enzyme (ACE)/angiotensin II (Ang II)/Ang II receptor type 1 axis and its counterpart ACE2/angiotensin-(1–7)/MAS1 receptor pathway as potential therapeutic targets. Given the lack of effective pharmacological ARDS treatments, clinical trials exploring new therapeutic strategies, like that by Khan et al. [1], are essential.

The association of ACE activity with ARDS outcomes is far from linear. Multiple factors, like the heterogeneity of ARDS and the variability of RAS activation in patient subgroups, could be implicated in this complex interplay [1]. Even though increased levels of ACE and Ang II have been detected in patients with ARDS, the pathophysiological role of RAS in ARDS development remains unclear. Additional factors such as, for example, the variable substrates of ACE and ACE2 apart from Ang (namely bradykinin and apelin), the protective effects against lung injury which Ang II receptor type 2 exerts, and the effect of ACE2 via the MAS1 receptor (and not via the Ang II receptor type 1) could be implicated [2]. There are encouraging data from animal models on the positive effects of ACE2 in ARDS, but respective data involving humans are insufficient [2].

In our study [3], higher ACE levels were not associated with the oxygenation index, even though they (but not the D/D genotype, which relates to higher serum ACE levels) seemed to correlate with ARDS prognosis. Zambelli et al. [4] showed improved arterial oxygenation (although not clinically relevant) in an experimental ARDS model after administration of angiotensin-(1–7), but no difference in survival or pulmonary mechanics. In Khan et al.’s study [1], administration of recombinant ACE2 in ARDS patients decreased Ang II levels without causing hypotension, but worsened respiratory mechanics. Khanna et al. [5] demonstrated in ATHOS-3 that Ang II administration in patients with vasodilatory shock increased blood pressure, without raising the risk for ARDS.

It is apparent that there are variable and often conflicting data in the current literature on the complex association between RAS and ARDS, which warrant the conduction of additional clinical trials to further elucidate its pathophysiological background and evaluate potential new treatments.

## Abbreviations

ACE: Angiotensin-converting enzyme
ACE2: Angiotensin-converting enzyme 2
Ang II: Angiotensin II
ARDS: Acute respiratory distress syndrome
RAS: Renin–angiotensin system
