# Supplementary material for: The evolving role of the renin–angiotensin system in ARDS
Source: Crit Care. 2017 Dec 28;21:329. doi: 10.1186/s13054-017-1917-5 (PMC5746002; doi:10.1186/s13054-017-1917-5)
Supplement: Related texts

# Reply: Authors’ response

**Authors:** Andrew I. Bayliffe, Akram Khan, Aili L. Lazaar

We are grateful for the comments of Vrigkou et al., and share their views regarding the need for more investigations into RAS biology in ARDS and other acutely ill patients. In addition to the well-documented effects on vascular tone and fluid/electrolyte homeostasis, the “classical” RAS system, through the action of Ang II and its receptor angiotensin receptor 1 (AT1R), elicits inflammation, oxidative stress, cellular apoptosis, and fibroproliferative responses [6], all important components of ARDS pathophysiology. ACE2 has been shown to improve both pathophysiology and outcomes in various animal models of ARDS by reducing Ang II/AT1R signaling *and* activating the regulatory angiotensin-(1–7)/Mas1 pathway, as demonstrated by studies that administer ACE2 or angiotensin-(1–7) peptide alone [7, 8].

In relation to the genetic evidence supporting a role for the RAS in human ARDS, we agree with Vrigkou et al. that the data are somewhat contradictory, probably because heterogeneity within ARDS cohorts hampers interpretation of genetic association studies. Despite this limitation, there are data from meta-analyses suggesting an association of the D-allele of the *ACE* gene with both ARDS risk [9] and mortality [3]. Together with work elucidating ACE2 as the receptor for the severe acute respiratory syndrome (SARS) coronavirus that caused uncharacteristically severe lung injury associated with profound depletion of ACE2 [10], these data suggest an important role for ACE2 in ARDS pathophysiology.

Our pilot clinical trial was designed to establish preliminary safety and tolerability, with an emphasis on monitoring hemodynamic stability, and to define exposure–response relationships with the RAS system. As mentioned in our discussion, readers should exercise caution when interpreting the “effects” of study treatments on physiology and respiratory mechanics given the small sample size and the risk of ascertainment bias at later time points, where only a small number of patients contribute to point estimates and significant imbalances between groups are apparent.

Finally, the contrast with the ATHOS-3 trial [5] where Ang II infusions elicited acute improvements in MAP in patients with high-output shock is important. Rather than revealing a novel protective role of Ang II via its other receptor AT2R, we believe this confirms the well-established vasopressor effects of Ang II, mediated via AT1R. To rationalise whether classical RAS signaling is beneficial or harmful in acutely ill patients, more trials with Ang II and ACE2 are required to fully understand their longer-term safety profile, their impact on RAS and other biological systems, and their impact on organ failure and outcomes. In the meantime, we speculate that there may be a place for both therapies in different patient populations, with short-term repletion of Ang II in certain vasoplegic shock patients with low Ang II levels, and repletion of ACE2 in ARDS patients with a high Ang II/angiotensin-(1–7) ratio.
